# The impact of *Mycobacterium tuberculosis* on the macrophage cholesterol metabolism pathway

**DOI:** 10.3389/fimmu.2024.1402024

**Published:** 2024-05-30

**Authors:** Zhanpeng Chen, Xingxing Kong, Quan Ma, Jinyun Chen, Yuqin Zeng, Huazhen Liu, Xiaomin Wang, Shuihua Lu

**Affiliations:** National Clinical Research Center for Infectious Diseases, Shenzhen Third People’s Hospital, Shenzhen, Guangdong, China

**Keywords:** MTB, TB, cholesterol metabolism, foam cell, macrophages, lipid droplet, oxidized cholesterol

## Abstract

Mycobacterium tuberculosis (Mtb) is an intracellular pathogen capable of adapting and surviving within macrophages, utilizing host nutrients for its growth and replication. Cholesterol is the main carbon source during the infection process of Mtb. Cholesterol metabolism in macrophages is tightly associated with cell functions such as phagocytosis of pathogens, antigen presentation, inflammatory responses, and tissue repair. Research has shown that Mtb infection increases the uptake of low-density lipoprotein (LDL) and cholesterol by macrophages, and enhances *de novo* cholesterol synthesis in macrophages. Excessive cholesterol is converted into cholesterol esters, while the degradation of cholesterol esters in macrophages is inhibited by Mtb. Furthermore, Mtb infection suppresses the expression of ATP-binding cassette (ABC) transporters in macrophages, impeding cholesterol efflux. These alterations result in the massive accumulation of cholesterol in macrophages, promoting the formation of lipid droplets and foam cells, which ultimately facilitates the persistent survival of Mtb and the progression of tuberculosis (TB), including granuloma formation, tissue cavitation, and systemic dissemination. Mtb infection may also promote the conversion of cholesterol into oxidized cholesterol within macrophages, with the oxidized cholesterol exhibiting anti-Mtb activity. Recent drug development has discovered that reducing cholesterol levels in macrophages can inhibit the invasion of Mtb into macrophages and increase the permeability of anti-tuberculosis drugs. The development of drugs targeting cholesterol metabolic pathways in macrophages, as well as the modification of existing drugs, holds promise for the development of more efficient anti-tuberculosis medications.

## 1 Introduction

Tuberculosis (TB) is a chronic infectious disease caused by *Mycobacterium tuberculosis* (Mtb). According to the World Health Organization, TB is the second leading cause of death from a single infectious agent worldwide in 2022, surpassed only by coronavirus disease 2019 (COVID-19). It is estimated that approximately one-quarter of the global population is infected with Mtb which typically leads to pulmonary tuberculosis. Without proper treatment, the mortality rate among TB patients can reach up to 50% (1). Mtb primarily invades the respiratory tract and spreads through airborne droplets to infect the lung tissues of the host. As a highly adaptable pathogenic bacterium, Mtb is capable of evading host immune system attacks through various mechanisms and establishing infection within host macrophages. Macrophages are critical components of the body’s immune system with the ability to engulf and degrade pathogens, thus playing a role in the clearance of infections. However, Mtb can adapt and survive within macrophages, utilizing host nutrients to provide energy and materials for its growth and replication (2).

Cholesterol is the main carbon source during the infection process of Mtb. Cholesterol is crucial for the optimal growth and persistence of Mtb. It serves as a critical growth substrate for Mtb within phagosomes and promotes the formation of foamy macrophages and necrotic granulomas. Mtb can utilize cholesterol as the sole carbon source for energy synthesis and membrane lipid production, leading to the generation of virulence lipids in its cell wall. Genes involved in cholesterol metabolism play a vital role in Mtb infection, and inhibition of the expression of these genes suppresses Mtb growth (3–5). Upon infection by Mtb, macrophages exhibit inhibition of cholesterol ester hydrolysis, facilitation of cholesterol esterification, hyperactivation of cholesterol synthesis metabolism, augmentation of cholesterol intake, and reduction of cholesterol efflux, which ultimately results in cholesterol accumulation. The excessive accumulation of cholesterol forms characteristic lipid droplet structures, promoting the formation of foam-like macrophages (6–9). The formation of foam cells not only suppresses the immune response of host cells but also helps Mtb achieve immune evasion and sustained survival, leading to the development of tuberculosis granulomas, tissue cavitation, and systemic dissemination of Mtb, which in turn contribute to the adverse progression of TB (10–12). Furthermore, cholesterol in macrophages can be converted into oxidized cholesterol which exhibits activity against Mtb infection (13).

In this review, we summarize the existing research findings, elucidate the significant role of cholesterol in macrophage function, and explore how Mtb regulates cholesterol metabolism pathways in macrophages, including cholesterol uptake, synthesis, esterification, hydrolysis, oxidation, and efflux. It would help to understand the pathological evolution process of TB and provide an important theoretical basis for the developing of new treatment strategies and vaccines, aiming to achieve the challenging goal of TB elimination by 2035.

## 2 Cholesterol and macrophage function

Macrophages, as immunocytes, play crucial roles in maintaining tissue homeostasis, host defense, and tissue repair in various physiological and pathological conditions. Cholesterol, a major lipid component of eukaryotic cell membranes, is vital for maintaining the stability of macrophage membranes and prominent impacts macrophage functions (14, 15). Cholesterol is a double-edged sword. On the one hand, it is indispensable for phagocytosis, antigen presentation, and other important functions of macrophages. On the other hand, excessive cholesterol accumulation impairs the tissue repair ability of macrophages and promotes foam cell formation.

**Figure 1:**
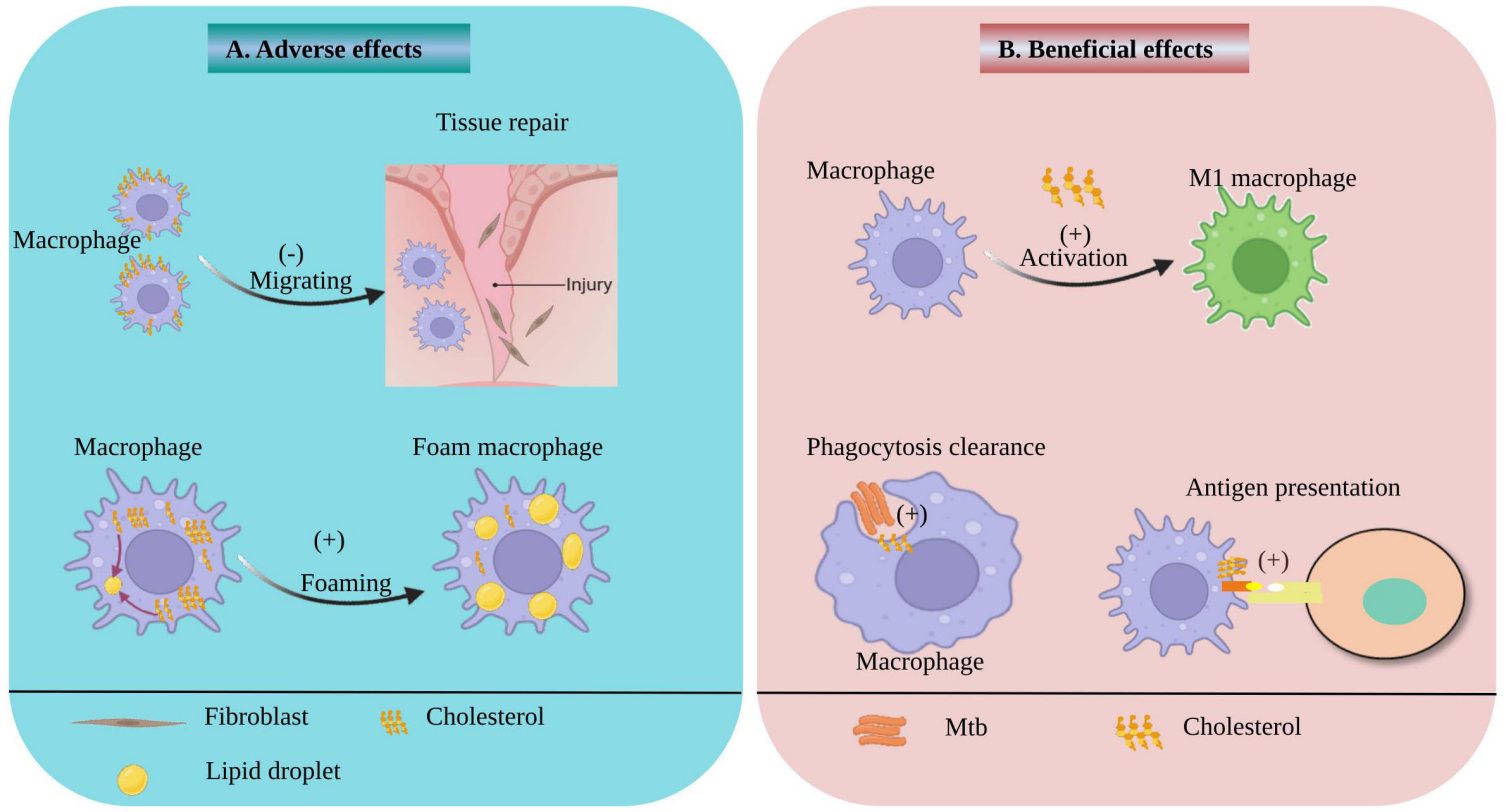
Beneficial and adverse effects of Cholesterol on macrophage. **(A)** the adverse effects of cholesterol on macrophage. The accumulation of cholesterol on macrophage membranes can inhibit macrophage migration and impede tissue repair. Cholesterol accumulation in macrophages promotes the formation of lipid droplets and foam cells. **(B)** the beneficial effects of cholesterol on macrophage. Cholesterol also can facilitate the activation of macrophages into M1 macrophages and promote macrophage antigen presentation. In addition, membrane cholesterol can enhance phagocytosis of Mtb by macrophages.

Macrophages play a key role in clearing pathogens from the body through phagocytosis. Studies have shown that cholesterol on the macrophage membrane acts as a signaling molecule, mediating macrophage phagocytosis of Mtb and facilitating Mtb entry into macrophages (16). Thus, cholesterol serves as a bridge for macrophage-mediated pathogen clearance. As an essential component of the cell membrane, cholesterol not only mediates the phagocytosis of macrophages but also strengthens its antigen presentation function. A study was conducted to evaluate the role of cholesterol in macrophage phagocytosis and antigen presentation by treating macrophages with a cholesterol biosynthesis inhibitor, and the effects were observed at different time points. The results indicated that cholesterol depletion inhibits the intracellular transport of major histocompatibility complex class I (MHC I) antigens, thereby reducing the surface expression level of MHC I molecules on macrophages (17). Another study also found that increased cholesterol levels may enhance antigen presentation by upregulating the expression of human leukocyte antigen class II (HLA-D) region products on antigen-presenting cells (18). It is well known that macrophages adopt different phenotypes, mainly divided into pro-inflammatory M1 macrophages and anti-inflammatory M2 macrophages, upon phagocytosis of pathogens or exposure to antigens. Accumulation of cholesterol in macrophages mainly promotes the generation of M1 macrophages, which enhance macrophage inflammatory responses. It has been demonstrated that Mtb infection promotes cholesterol accumulation in macrophages, which may further activate the NOD-like receptor family pyrin domain containing 3 (NLRP3) inflammasome, leading to increased production of cytokines such as interleukin (IL)-1β, IL-6, and tumor necrosis factor (TNF)-α, as well as reactive oxygen species (ROS) (14, 19). In addition, cholesterol not only serves as a pro-inflammatory mediator but also constitutes an important structural domain for catalyzing immune responses. Studies have shown that cholesterol-rich domains on the membrane regulate immune responses by modulating the nuclear transport of interferon-gamma (IFN-γ) and interferon gamma receptor 1(IFNGR-1), as well as the activation of signal transducer and activator of transcription 1 alpha (STAT1α) (20). On the basis of the inflammatory phenotype, macrophages also exhibit a pathological phenotype known as foam cell phenotype, which plays a crucial role in the development of TB. Research has demonstrated that Mtb promotes its survival by reprogramming the metabolism of host macrophages, leading to the accumulation of cholesterol as a carbon source for its own survival. Simultaneously, Mtb-mediated cholesterol accumulation further promotes the formation of foam cells (6, 21). Foam cells serve as a sanctuary for Mtb survival and are also a hallmark of TB lesions. Foam cells formed during Mtb infection are not only key participants in granuloma formation and tissue cavitation, but they also further facilitate systemic dissemination of Mtb upon their departure from the primary granuloma (10–12, 22). Macrophages not only possess phagocytic and immune response functions but also play a role in tissue repair. It is well-known that tissue repair requires recruitment of a large number of macrophages. Studies have shown that cholesterol can regulate cell stiffness and motility, and excessive cholesterol accumulation inhibits the chemotactic migration of macrophages to wounds, thereby impairing wound healing (23). Interestingly, Mtb-infection can induce the production of oxidised cholesterol, thereby enhancing the host’s immune response to Mtb-infection (24). In conclusion, cholesterol plays a crucial role in macrophage pathogen engulfment, antigen presentation, and inflammatory responses. It is closely associated with the development of TB, and also affects the tissue repair function of macrophages (**Figure 1**).

## 3 The effect of Mtb infection on macrophage cholesterol metabolism

### 3.1 Cholesterol intake

Cholesterol can exist in the form of free cholesterol, but it is more commonly transported to tissues throughout the body, including macrophages, in the form of low-density lipoprotein (LDL). Macrophages uptake cholesterol and its lipoproteins through a family of low-density lipoprotein receptor (LDLR) proteins expressed on cell membranes. Low-density lipoprotein receptor-related protein 2 (Lrp2), a 600 kDa giant membrane glycoprotein, belongs to the LDLR protein family and is primarily responsible for mediating the uptake of intracellular cholesterol. Lrp2 expression was found to be remarkably increased in macrophages infected with H37Rv, and it is positively correlated with the accumulation of intracellular cholesterol (7). Cluster of differentiation 36 (CD36), a transmembrane glycoprotein belonging to the LDLR protein family, forms a channel through the cell membrane and is responsible for mediating the absorption of lipid molecules. Tuberculous pleural effusion has been shown to induce massive accumulation of cholesterol in macrophages and markedly enriches the fluorescence intensity of CD36 on the cell membrane (25). Further evidence from a guinea pig model of Mtb infection confirms the accumulation of oxidized low-density lipoprotein (OxLDL) in extrapulmonary granulomas and pulmonary macrophages of TB guinea pigs. Moreover, macrophages increase the surface expression of scavenger receptor CD36 and lectin-like oxidized low-density lipoprotein receptor-1 (LOX1), promoting the uptake of cholesterol and LDL (26). LOX1 is a membrane receptor protein belonging to the C-type lectin family involved in the absorption and transport of LDL. A study was conducted to analyze granuloma samples from human TB. Transcriptomic analysis revealed a significant upregulation of genes involved in cholesterol and other lipid synthesis, and immunohistochemistry confirmed the high expression of these lipid synthesis proteins in human TB granulomas. Thin-layer chromatography and mass spectrometry analyses revealed that granulomas and foam cells were rich in cholesterol, cholesterol esters, and triglycerides, with LDL being a probable major source of these lipids (9).

**Figure 2:**
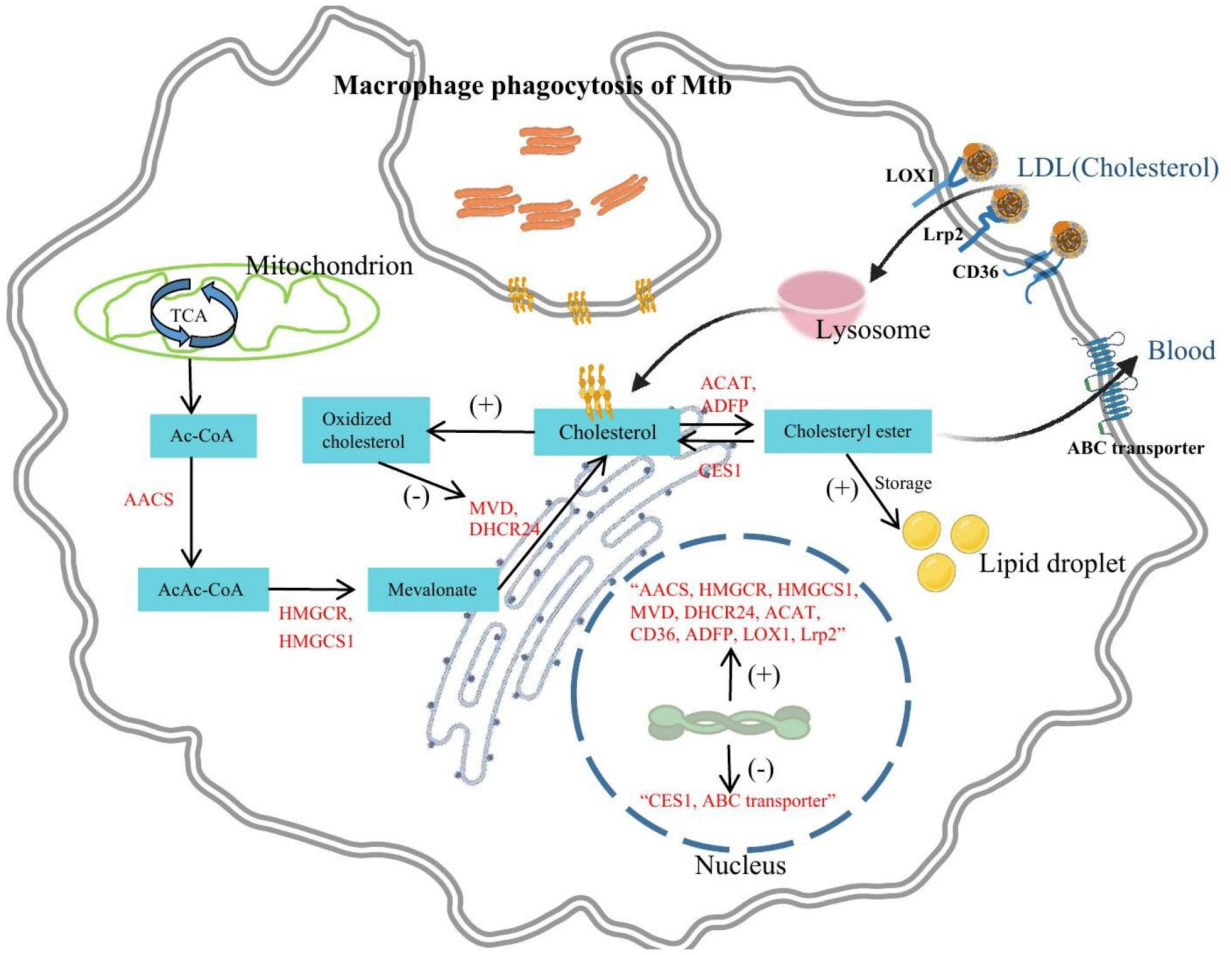
The effects of Mtb infection on macrophage cholesterol metabolism. Mtb infection significantly impacts macrophage cellular processes. It increases the uptake of LDL and cholesterol through upregulation of receptors like Lrp2, CD36, and LOX1. Additionally, Mtb infection stimulates *de novo* cholesterol synthesis by upregulating enzymes like AACS, HMGCS1, HMGCR, MVD, and DHCR24. This excess cholesterol is converted into cholesterol esters, contributing to lipid droplet formation facilitated by upregulation of ADFP and ACAT. Cholesterol ester degradation is hindered due to downregulation of CES1. Mtb infection also suppresses ABC transporters, impeding cholesterol efflux. These alterations lead to cholesterol accumulation, ultimately promoting the formation of foam cells. Furthermore, cholesterol in macrophages may also be converted into oxidized cholesterol which exhibits anti-Mtb activity.

In summary, cholesterol is primarily taken up by macrophages in the form of LDL in the body. Macrophages uptake cholesterol and LDL through the expression of LDLR family proteins, such as Lrp2, LOX1, and CD36. Although animal experiments and human samples provide some evidence, further clinical research is needed to validate these findings, especially in observing the accumulation of cholesterol and LDL and the expression changes of receptors in the human body during Mtb infection. Besides, other potential receptors involved in the uptake of cholesterol and LDL, as well as their roles in the development of the disease, can be explored to provide new therapeutic strategies for the prevention and treatment of TB (**Figure 2**).

### 3.2 Cholesterol synthesis

Multiple transcriptomic studies have revealed that Mtb infection leads to upregulation of cholesterol synthesis-related enzyme gene expression in macrophages, including acetoacetyl-coenzyme A synthetase (AACS), 3-hydroxy-3-methylglutaryl- coenzyme A synthase 1 (HMGCS1), 3-hydroxy-3-methylglutaryl-coenzyme A reductase (HMGCR), mevalonate diphosphate decarboxylase (MVD), and 3β-hydroxysterol Δ24-reductase (DHCR24) (7, 27). AACS is a cytoplasmic enzyme involved in fatty acid biosynthesis that catalyzes the coupling of acetyl-coenzyme A (CoA) to produce acetoacetyl-CoA, which serves as the starting substrate for cholesterol synthesis (28). Therefore, the promotion of AACS gene expression by Mtb may contribute to *de novo* cholesterol synthesis. HMGCS1 is a key enzyme in the mevalonate pathway of cholesterol biosynthesis, catalyzing the conversion of acetoacetyl-CoA to 3-hydroxy-3-methylglutaryl-CoA (HMG-CoA), which is further reduced to mevalonic acid by HMGCR, a rate-limiting enzyme in cholesterol synthesis that regulates cholesterol production (29). HMGCR and HMGCS1 play pivotal roles in macrophage function. According to previous research, HMGCR knockout was found to significantly inhibit the migration, proliferation, and survival of macrophages. However, another study found that increased expression of HMGCR induces excessive cholesterol synthesis, resulting in decreased membrane fluidity and suppressed phagocytic activity of macrophages (30, 31). When HMGCS1 activity is excessively elevated, it can cause the accumulation of hydroxymethylglutaryl-CoA in macrophages, which result in cell necrosis. Necrosis is a unique form of cell death characterized by cell membrane rupture, cytoplasmic leakage, and organelle damage. In macrophages, cell death induced by HMGCS1 via necrosis pathway exerts irreversible effects on the physiological function of macrophages, thereby impacting the normal immune system function (32). Furthermore, HMGCR and HMGCS1 are key genes in promoting macrophage foam cell formation. Increased activity of HMGCR and HMGCS1 contributes to excessive activation of the cholesterol synthesis pathway, which in turn leads to macrophage lipid droplet formation (33). Therefore, Mtb-induced overexpression of HMGCR and HMGCS1 may inhibit phagocytic activity of macrophages, promote cholesterol accumulation and foam cell formation, and even cause macrophage death.

MVD catalyzes the conversion of mevalonate diphosphate to isopentenyl diphosphate, which is an early step in cholesterol biosynthesis (34). MVD and the mevalonate pathway are crucial for macrophage survival and play an important role in the synthesis and release of inflammatory mediators, contributing to pro-inflammatory responses of macrophages (35). DHCR24, also known as 3β-hydroxysterol Δ24-reductase, is a human flavin adenine dinucleotide (FAD)-dependent oxidoreductase that catalyzes the final step of cholesterol synthesis, converting desmosterol to cholesterol (36). In addition to HMGCR and HMGCS1, DHCR24 is also a key gene in promoting macrophage foam cell formation and possesses antioxidant functions that effectively inhibit oxidative stress (33, 37). Consequently, Mtb infection of macrophages may elevate immune inflammation levels, inhibit oxidative stress, and induce cholesterol accumulation as well as foam cell formation by upregulating MVD and DHCR24 expression. Despite some progress has been made in current studies, most of them have been conducted using *in vitro* cell culture models, and these findings need to be further validated *in vivo*. Animal models or clinical samples can provide a better understanding of the impact of Mtb infection on cholesterol anabolism in macrophages (**Figure 2**).

### 3.3 Esterification of cholesterol

Accumulation of cholesterol esters (CE) stored in cytoplasmic lipid droplets is a key feature of foam cells, which are central to the development of atherosclerosis and tuberculosis (38). When macrophages are infected with Mtb, not only does it promote cholesterol accumulation, but it also promotes the conversion of cholesterol to cholesterol esters within macrophages, thereby facilitating the formation of lipid droplets and foam cells. Animal pathology studies have shown that macrophages isolated from granulomatous lungs of tuberculosis mice contain large amounts of cholesterol esters, whereas the ester content in resident peritoneal macrophages of normal mice is very low (39). Metabolomics studies of human lung tissue have revealed that large amounts of cholesterol, cholesteryl esters, and triglycerides accumulate in the granulomas of patients with TB. All of these studies provide ample evidence that cholesterol in Mtb-infected macrophages is converted to cholesterol esters for storage. Further mechanistic exploration has revealed that Mtb, especially its cell wall surface lipid trehalose dimycolate, upregulates the expression of adipose differentiation-related protein (ADRP), thereby promoting cholesterol esterification and lipid droplet formation (9). Acyl-CoA: cholesterol acyltransferase (ACAT) is a key enzyme catalyzing cholesterol esterification in macrophages, and it has been found that tuberculosis pleural effusion (TB-PE) induces the expression of ACAT, promoting cholesterol esterification in macrophages. When ACAT is inhibited, the levels of cholesterol esters and triglycerides in TB-PE-treated macrophages are significantly reduced (25). Accordingly, it can be concluded from the existing studies that Mtb and its cell wall surface lipid virulence factors enhance the conversion of cholesterol to cholesterol esters in macrophages by upregulating the expression of ACAT and ADFP, thereby facilitating the formation of lipid droplets and foam cells. Currently, there are still some research gaps and directions that need further exploration. Firstly, the detailed mechanisms underlying the accumulation of cholesterol esters in Mtb-infected macrophages have not been fully understood, including how Mtb regulates the expression and activity of ACAT. In the future, more animal models and patient samples should be utilized to further explore the mechanisms of the accumulation of cholesterol esters in Mtb-infected macrophages (**Figure 2**).

### 3.4 Hydrolysis of cholesterol esters

Only non-esterified or free cholesterol (FC) can efflux from the cell to the extracellular space, thus the excessively accumulated cholesterol esters need to be effectively degraded by cells to prevent pathological progression. Cholesterol esters are hydrolyzed by cholesterol esterase within the cells, resulting in the formation of free cholesterol and fatty acids (38). Cholesterol ester hydrolysis contributes to the reduction of lipid load in macrophages, diminishing inflammation and related diseases, and is crucial for macrophage function (40). Inhibition of cholesterol ester hydrolysis leads to dysregulation of cholesterol content in microdomains, which prolongs the activation of immune cell signaling pathways and promotes immune cell proliferation, infiltration, and foam cell formation (41). A study using transcriptomic analysis and *in vitro* validation demonstrated that macrophage receptor with collagenous structure (MARCO) and collectin sub-family member 12 (COLEC12), which are involved in phagocytosis, as well as carboxylesterase 1 (CES1), the cholesterol ester hydrolase, were downregulated in alveolar macrophages (AMs) from TB patients compared to controls, indicating impaired cholesterol ester degradation and reduced free cholesterol, leading to excessive accumulation of cholesterol esters and adverse progression of pulmonary TB (8). Further research can further validate the key role of CES1 in TB progression through protein detection and functional experiments, and explore the specific mechanisms by which Mtb regulates the downregulation of macrophage CES1 using cell and animal models (**Figure 2**).

### 3.5 The oxidation of cholesterol

In addition to cholesterol esters, cholesterol can also be converted into oxidized cholesterol forms in macrophages, with 25-hydroxycholesterol(25HC)and 1,25 dihydroxyvitamin D (1,25(OH)_2_D) being two typical oxidized cholesterol derivatives. Mtb infection induces macrophage activation, and activated macrophages are a rich source of oxidized cholesterol (42–45). In macrophage models of Mtb infection, oxidized cholesterol typically exerts an antimicrobial effect. Studies have shown that the production of 25HC is significantly increased in activated macrophages, which in turn inhibits cholesterol biosynthesis and accumulation, while cholesterol accumulation promotes the growth of Mtb (21, 46). An increase in 25HC concentrations was shown in lungs of mice infected with Mtb (24). Furthermore, 7α,25-dihydroxycholesterol can increase Mtb phagocytosis and reduces intracellular Mtb growth in primary human monocytes (47). Multiple studies have demonstrated the potent anti-Mtb infection activity of 1,25(OH)_2_D (48). 1,25(OH)_2_D inhibits Mtb growth by suppressing cholesterol and lipid droplet formation (49). Additionally, 1,25(OH)_2_D promotes the production of antimicrobial peptides and the maturation of phagolysosomes in macrophages, thereby enhancing an effective cellular immune response against Mtb (13, 50). In conclusion, Mtb infection may promote the conversion of cholesterol into oxidized cholesterol in macrophages, and oxidized cholesterol plays a promoting role in macrophage-mediated anti-Mtb infection. Future research needs to explore the underlying mechanisms and target points by which Mtb infection promotes the production of oxidized cholesterol, as well as develop new therapeutic strategies for anti-Mtb infection based on oxidized cholesterol (**Figure 2**).

### 3.6 The efflux of cholesterol

Even though cholesterol esters can be degraded to cholesterol, macrophages themselves are unable to degrade cholesterol. Therefore, when cholesterol accumulates excessively in macrophages, it ultimately needs to be eliminated from the cells through efflux. ATP-binding cassette (ABC) transporters are a class of transmembrane proteins that utilize ATP hydrolysis to mediate cholesterol efflux, including genes such as ABCA1, ABCG1, and ABCA5 (51). *In vitro* studies have shown that infection of macrophages with the Mtb strain H37Ra inhibits the expression of ABCG1 and promotes the formation of lipid bodies and foam cells. In contrast, overexpression of ABCG1 in macrophages through lentiviral transduction can significantly reverse foam cell formation (52). Researchers have constructed a macrophage model infected with Bacille Calmette-Guérin (BCG) and found that BCG infection inhibit the expression of macrophage ABCG1, ABCA1, and ABCA5, promoting intracellular cholesterol accumulation and further inducing macrophage autophagy to suppress BCG infection (53). Further animal experiments have provided more compelling evidence, as it was found that the expression of ABCA1 and ABCG1, which are involved in cholesterol efflux, was significantly down-regulated in peritoneal macrophages and lung tissue sections of mice infected with H37Ra (7). In conclusion, Mtb infection has a direct regulatory effect on cholesterol efflux in macrophages by downregulating the expression of ABC transporters, inhibiting cholesterol efflux, and promoting cholesterol accumulation and foam cell formation. Current research mainly focuses on *in vitro* experiments and animal experiments, and further human-related studies, including clinical research and proteomic studies, are needed to assess the impact of Mtb infection on ABC transporters and cholesterol metabolism, as well as to explore the relevant molecular mechanisms. Additionally, while overexpression of ABC transporters can reverse foam cell formation, further studies are needed to investigate their potential therapeutic efficacy in the treatment of TB (**Figure 2**).

## 4 Foamy macrophage and TB progression

Foam cells are lipid-rich macrophages that serve as a hallmark of granulomas and tuberculous lesions in TB. Consistent with the discussion in the previous section, Mtb infection promotes the accumulation of lipids such as cholesterol in macrophages, leading to the formation of foam macrophages (54). Foam cells exhibit various functions in tuberculosis. They possess anti-apoptotic properties, as evidenced by the upregulation of anti-apoptotic protein expression, which prevents cell death (55, 56). Additionally, foam cells exhibit dormancy capabilities. Research has shown that Mtb remains in a non-replicating state within foam cells, facilitating persistent infection (57). Furthermore, foam cells exert anti-inflammatory functions in TB. Mtb-infected foam cells decrease secretion of TNF-α and IL-1α, increase secretion of Transforming Growth Factor (TGF)-β, and inhibit effector T cells through inducing higher levels of nitric oxide (58, 59). Foam cells not only undergo functional changes but also play a role in promoting TB progression. Firstly the lipid-rich content of foam cells provides abundant nutrients and a stable environment for intracellular growth of Mtb (11). Moreover, foam cells play a central role in the development, maintenance, and dissemination of TB granulomas. Following Mtb infection, alveolar macrophages migrate to the interstitium and induce an inflammatory response, that leads to immune cell infiltration. These infiltrating immune cells surround foam cells which serve as reservoirs for Mtb, resulting in the formation of TB granulomas (60–62). The necrotic area surrounding the lipid-rich core is referred to as caseation. Foam cells promote the formation of the necrotic core by releasing their abundant content of triglycerides into the caseum, leading to proteinaceous transformation and enlargement of granulomas, ultimately causing progressive destruction of lung tissue and loss of pulmonary function (63). Foam cell death or departure from the primary granuloma generates secondary granulomas, facilitating the systemic dissemination of Mtb (11, 64).

Not only macrophage foam formation, but also macrophage phenotypes play a crucial role in the progression of tuberculosis. During the early stages of Mtb infection, macrophages differentiate into M1 macrophages (65). M1 macrophages exhibit the Warburg effect, promoting pro-inflammatory responses and enhancing ROS production, which helps restrict Mtb survival (66–68). Over time, the major virulence factor of Mtb, ESAT-6, converts M1 macrophages to M2 macrophages (69). M2 macrophages secrete IL-10 and TGF-β, inhibiting antimicrobial immunity and promoting sustained Mtb survival (70, 71). Thus, further investigation into the transition process from M1 to M2 macrophages induced by Mtb can reveal potential immune targets for TB. We should also pay particular attention to and explore the role of cholesterol metabolism during this transitional process.

## 5 Summary

During the occurrence and development of TB, Mtb infection exerts multiple impacts on the cholesterol metabolism in macrophages. At first, Mtb infection promotes the uptake of LDL and cholesterol accumulation through receptors expressed on macrophages, such as Lrp2, LOX1, and CD36. Secondly, Mtb infection leads to upregulation of genes involved in cholesterol synthesis enzymes (AACS, HMGCS1, HMGCR, MVD, and DHCR24), enhancing cholesterol synthesis. Also, Mtb infection promotes the conversion of cholesterol to cholesterol esters, increasing lipid droplet accumulation. However, Mtb infection may inhibit the expression of CES1, which result in excessive accumulation of cholesterol esters and the formation of adverse foam cells. Furthermore, Mtb infection inhibits the expression of ABC transporters in macrophages, impeding cholesterol efflux and further exacerbating the generation of adverse foam cells. While oxidized cholesterol demonstrates good activity in combating Mtb infection, the mechanisms by which Mtb infection impacts oxidized cholesterol generation remain unclear. In summary, the dysregulation of cholesterol metabolism in macrophages caused by Mtb infection is an important feature in the development of TB, and it is of great significance for a deeper understanding of the pathogenesis of TB and the search for therapeutic strategies.

Currently, there are drug developments targeting the cholesterol metabolism pathway for the treatment of TB. For example, statins reduce cholesterol levels in macrophages by inhibiting HMGCR, thereby reducing the entry of Mtb into macrophages and improving the permeability of anti-tuberculosis drugs (72). Although negative results exist in certain situations, statin therapy as an adjunctive treatment has demonstrated improvements in the treatment outcomes of TB patients in clinical practice (11). Therefore, it is promising to develop effective anti-tuberculosis drugs by repurposing existing drugs. GSK2556286 is a novel anti-tuberculosis drug targeting cholesterol metabolism, which inhibits cholesterol catabolism by acting on adenosine nucleotide cyclase, thereby suppressing the intracellular and extracellular growth of Mtb (73). In the early stages of clinical trials, it has demonstrated the potential to shorten TB treatment and can be considered as a potential substitute for pretomanid or linezolid in the BPaL regimen. Moreover, it does not exhibit cross-resistance with known anti-tuberculosis drugs (74, 75). 1,25(OH)_2_D exhibits excellent anti-tuberculosis activity, and related clinical trials have shown that vitamin D may help minimize excessive tissue damage during active TB (76). Therefore, further investigation and application of oxidized cholesterol are warranted. For detailed information on the association between lipid metabolism and anti-tuberculosis drugs, please refer to another review article (77). In the future, we should not only further validate and explore the regulatory mechanisms of Mtb infection on cholesterol metabolism in macrophages, but also focus on the key targets of cholesterol metabolism pathways during Mtb infection to design efficient anti-tuberculosis drugs.

## Author contributions

CZ: Conceptualization, Writing – original draft, Writing – review & editing. XK: Writing – review & editing. QM: Writing – review & editing. JC: Writing – review & editing. YZ: Writing – review & editing. HL: Writing – review & editing. XW: Conceptualization, Supervision, Writing – review & editing. SL: Conceptualization, Supervision, Writing – review & editing.
